# Coupling tensile test with LC-OCT and ultrasound imaging: investigation of the skin sublayers mechanical behaviour

**DOI:** 10.1098/rsos.231712

**Published:** 2024-06-12

**Authors:** Ianis Ammam, Amaury Guillermin, Lucas Ouillon, Roberto Vargiolu, Jean-Luc Perrot, Hassan Zahouani

**Affiliations:** ^1^ Université de Lyon, ENISE, LTDS, UMR 5513 CNRS, 58 rue Jean Parot, Saint-Etienne Cedex 2 42023, France; ^2^ Dermatology Department, University Hospital of Saint-Etienne, Saint-Etienne, France

**Keywords:** skin, viscoelastic properties, multi-layer behaviour, skin anisotropy, imaging tools

## Abstract

The skin is an envelope that covers the entire body. Nowadays, understanding and studying the mechanical, biological and sensory properties of the skin is essential, especially in dermatology and cosmetology. The in-depth study of the skin’s mechanical behaviour is a highly intriguing challenge, enabling the differentiation of the behaviour of each layer. An extension device was developed to perform relaxation and extension tests to characterize the skin. The device has also been coupled with imaging tools (LC-OCT and ultrasound), allowing us to observe layer-by-layer deformations during the tests. Relaxation tests revealed significant skin anisotropy, as well as an influence of age and gender on skin viscoelastic parameters calculated from relaxation curves and a skin viscoelastic model. These tests also unveiled their ability to distinguish certain characteristic pathologies that alter the mechanical properties of the skin, such as scleroderma or heliodermatitis. Furthermore, the optical–mechanical coupling and deformation calculation through image analysis demonstrated that the skin layers exhibit distinct mechanical behaviours owing to their different structures. Finally, Poisson’s ratio of the skin was obtained by calculating the deformation in two directions for each layer.

## 1. Introduction

The skin is the first border between our body and the outside world. It is a physical, chemical, immune and mechanical barrier against external aggressions [1]. The skin supports all the organs, resists shocks, is both permeable and impermeable, and plays a role in healing and temperature regulation [1]. Paradoxically, skin tissue also provides an opening to the world, as tactile perception is an extraordinary means of communication [1]. One of today’s challenges is to study and understand the mechanical, biological and sensorial behaviour of the skin. In dermatology, the practitioner assesses lesions by touch [2]. Thus, the use of a device to assess the mechanical properties of the skin will assist and complement the diagnoses of clinicians in an objective manner. In cosmetology, the mechanical study of the skin makes it possible to objectively analyse the impact of a product on it [1]. It is therefore very interesting to develop systems that translate the language of the skin by characterizing its mechanical properties. To this end, many quasi-static and dynamic mechanical tests have been developed: perpendicular to the skin such as suction [2–7], indentation [2,8,9] and indentation without contact [10,11]; and normal to the skin, torsion [1–3] and extension [1,2,12–15]. The extension test is a valuable technique for characterizing the skin mechanically. It consists of pulling the skin between two pads, one mobile and one fixed. It is a directional test that highlights skin phenomena that have been presented in the literature for many years [1,16–19] such as anisotropy, nonlinearity and viscoelasticity. However, this type of test on the skin *in vivo* is poorly defined, since neither the boundary conditions nor the initial conditions are known, and they are highly dependent on the area studied and the individual [13,14]. Furthermore, skin extension tests only allow the study of surface mechanical properties, as the applied force acts solely at the surface. However, the skin is a complex organ, composed of stratified layers with distinct structural characteristics. Many pathological conditions, nevertheless, affect the deeper layers of the skin. Therefore, it is interesting to investigate the mechanical behaviour of the skin in depth and to be able to differentiate the behaviour of each layer.

The aim of this study was to develop an extension device to perform *in vivo* skin relaxation tests. Additionally, we coupled this mechanical device with imaging tools, allowing us to investigate the behaviour of different skin layers through image analysis. This article presents how we studied and analysed the mechanical behaviour of the skin. Firstly, the tools that were used and/or developed, as well as the protocol used to perform the measurements, are presented. Then, the results obtained are presented and discussed. These results are divided into the following two parts: the general behaviour of the skin through the study of mechanical parameters, and multi-layer mechanical behaviour by mapping the mechanical behaviour of the skin layer by layer. This article presents the method developed for this study and the results obtained.

## 2. Material and methods

### 2.1. Extension device

**Figure 1.**
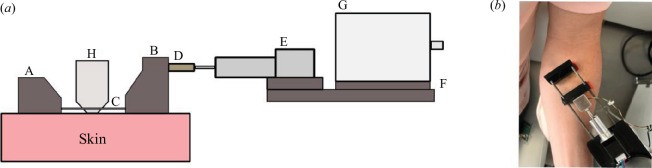
(*a*) Schematic of the traction device during an extension–imaging coupling (corresponding to the protocol given in §2.2.2). (*b*) Traction device in relaxation test performance on a forearm at 45° (corresponding to the protocol §2.2.1).

The extension device developed, shown in figure 1, is composed of two pads: one fixed (A) and one mobile (B) of equal size: 14 × 42 mm^2^. They are attached to the skin with double-sided hypoallergenic adhesive tapes (Monaderm, Monaco). Only the pads are in contact with the skin to avoid altering the natural state of the skin as much as possible. The area of stretched skin will therefore vary according to the distance between the two pads. In this study, the distance between the two pads at rest was 20 mm. This distance was chosen so as not to make the tests painful for the subjects but also to use the probes of the imaging devices.

The two pads are guided by two aluminium guide pins (C). The mobile pad is attached to an S-shaped load cell (D). This sensor has a maximum load of 1 kg with a sensitivity of 0.01 N. The sensor calibration was performed using several mechanical weights to calculate the sensor gains (10, 20, 50, 100 and 200 g). To validate the linearity, we measured the output voltage of the sensors for each mechanical weight. These measurements were performed five times for each mass to ensure repeatability.

The displacement of the mobile pad slightly compresses the sensor, allowing us to measure the tangential force on the skin. The movement of the pad is ensured by a Nema 8, LGA20 linear motor (Nanotec, Germany) (E). The motor is attached to the other end of the force sensor and to a frame (F). The frame is attached to the head of a Universal robot of the UR3e series (Universal Robot, Denmark) (G). The robot has six degrees of freedom. As the device is fixed on its head, it can move in all directions and on all areas. Finally, the probes of the imaging devices (Line-field confocal optical cohérence tomography (LC-OCT) and ultrasound) (H) are positioned between the device’s pads to measure the deformation of the skin in depth.

### 2.2. Measurement protocols

#### 2.2.1. Relaxation test

The relaxation tests were carried out on 12 volunteers (*n* = 12), six women and six men. Of the 12 volunteers, six were under 30 years old (24.6 years ± 2.1) and six were over 30 years old (46.6 years ± 7.5). Twelve subjects were chosen to have a parity of men and women and enough subjects to consider this work as a pre-study. However, this work was carried out during the spring of 2021, so in the context of the health crisis, it was impossible to have more than 12 subjects. A more comprehensive study could be conducted with more than 12 subjects. The tests were conducted in a controlled environment with a relative humidity of 30% and a temperature of 20°C. Finally, ethical consent to use the results was obtained for each volunteer in this study in accordance with the Declaration of Helsinki guidelines. Subjects were required to maintain intact skin without the addition of cosmetic or pharmaceutical products that could influence the mechanical properties of the skin.

**Figure 2.**
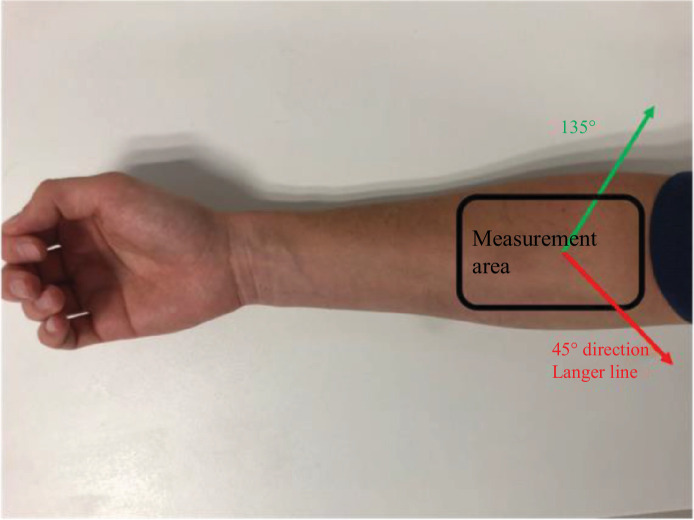
Measurement region on the forearm with the applied stress directions used for traction testing.

The volunteers had healthy skin, and the test area did not show any lesions. The test area was the inner forearm. The tests were carried out sitting with the arm positioned on an armrest, the hand holding a foam ball to limit movements as much as possible. The tests were performed in two directions (figure 2): 45° along the Langer lines, and 135° orthogonal to the Langer line, according to Ridge and Wright [20]. However, orientations close to that of Langer, for us 45°, were selected because Langer lines can vary from one individual to another, necessitating the fixation of a specific direction. For the second orientation, an orthogonal angle, which is 135°, was chosen. The objective was to study two distinct fibre families located near the direction of Langer.

The following parameters were used: speed: 1 mm s^−1^; initial length between the pads: 20 mm; strain: 20%; relaxation time: 30 s; number of cycles: 3. These parameters were determined in this manner to ensure that the tests were not painful and that there were sufficient data to study the curves. Moreover, 20% deformation allows us to observe the bi-linearity of the mechanical behaviour of the skin, below 20% or even 15% the deformation is too low to observe this phenomenon. Finally, the 30 s of relaxation allows us to have enough data to fit this part.

We also carried out this same protocol (only at 45°) on two women (*n* = 2 and older than 50 years) suffering from skin pathologies. A woman suffering from a crest, which corresponds to excess fibre and therefore abnormally rigid skin (like scleroderma). Another woman suffering from heliodermatitis, which corresponds to skin ageing and thus tissue relaxation. These patients were diagnosed by Professor Jean-Luc Perrot of the Dermatology Department, University Hospital of Saint-Etienne. In this scenario, Professor Jen-Luc Perrot diagnosed two skin conditions that exhibit completely different symptoms. The purpose was to assess whether our method could effectively distinguish between these two disparate skin pathologies.

#### 2.2.2. Extension–imaging coupling

For the extension–imaging coupling, the measurements were carried out at the University Hospital of Saint-Etienne on the forearm (*n* = 1 and man less than 30 years) following the same approach as for the relaxation tests for each imaging tool. The tests were performed in the following three directions (figure 2): along the Langer lines, perpendicular to the Langer line and in the arm axis, according to Ridge and Wright [20].

To study the skin in depth during an extension, the device was coupled to an Line-field confocal optical cohérence tomography device (LC-OCT) (Damae, France) and an ultrasound scanner (Vevo MD; Fujifilm SonoSite, USA), both available at the hospital.

To study the skin surface during an extension, we reproduced the skin relief with Silflo (Monaderm) prints, after which they were studied with a confocal microscope (Altimet, France). To reproduce the relief, we use Silflo, which is a silicone paste that cures with the addition of a catalyst. By applying the mixture to the area to be studied, here the forearm, the silicone solidifies and reproduces the skin relief at the time.

By analysing with the confocal microscope, it is possible to obtain very interesting images of this relief and to study them by image analysis.

### 2.3. Numerical analysis

As part of the numerical analysis, it is necessary to work in terms of stress/strain rather than force/displacement. Thus, the elastic modulus and rheological parameters were calculated in this context. The cross-sectional area of the skin on which the applied force acts was used to convert the forces applied in strain. Specifically, it is relevant to liken this extension test to a tensile test.

The cross-sectional area was calculated from the width l corresponding to the size of the pads ( l=42mm ; §2.1) and the skin thickness e affected by the extension. An extension test coupled with ultrasound and LC-OCT imaging was conducted to observe the thickness e [21–24]. This thickness is 1.2 mm, down to the hypodermis.

Hence, the effective cross-sectional area *S* is calculated as follows: S=e⋅l=50.4mm².

Now that we know the cross-sectional area *S* of the skin, the stress *σ* is defined based on the engineer’s stress expression as follows:


(2.1) $$\sigma =\frac{F_{mes}}{S}\;,$$

where $F_{mes}$ is the measured force.

In this section, a purely elastic context of the skin was assumed, and therefore Hooke’s law can be applied.

#### 2.3.1. Analysis of rheological parameters

Skin is a viscoelastic material that can be modelled with a generalized second-order Maxwell rheological model. To characterize the viscoelastic properties of the skin, a relaxation and creep curve fitting algorithm has been developed in the laboratory based on the studies discussed in [25–27].

The algorithm is based on a method in which the constitutive equations of such a model can be expressed, in part by a Prony series. Thus, the stress state for a relaxation test can be expressed according to Chen *et al.* [25]


(2.2) $$\sigma \left(t\right)=Y\left(t\right)\epsilon_{0}+\int_{0}^{t}Y\left(t-\zeta \right)\frac{d\epsilon \left(\zeta \right)}{d\zeta }d\zeta \;,$$

where $\int_{0}^{t}Y\left(t-\zeta \right)\times \frac{d\epsilon \left(\zeta \right)}{d\zeta }d\zeta$ considers the subsequent deformation at t ,

and $Y\left(t\right)$ is the relaxation function and is expressed in terms of a Prony series


(2.3) $$Y\left(t\right)=E_{0}\times \left(1-\sum_{i\;=\;1}^{N}p_{i}e^{-t/\tau_{i}}\right)\;,$$

where *i* corresponds to the number of branches in the model,

$p_{i}$ is the parameter of the Prony series,

$\tau_{i}$ is the relaxation time of the Prony series, and

$E_{0}$ is the instantaneous elastic modulus.

**Figure 3.**
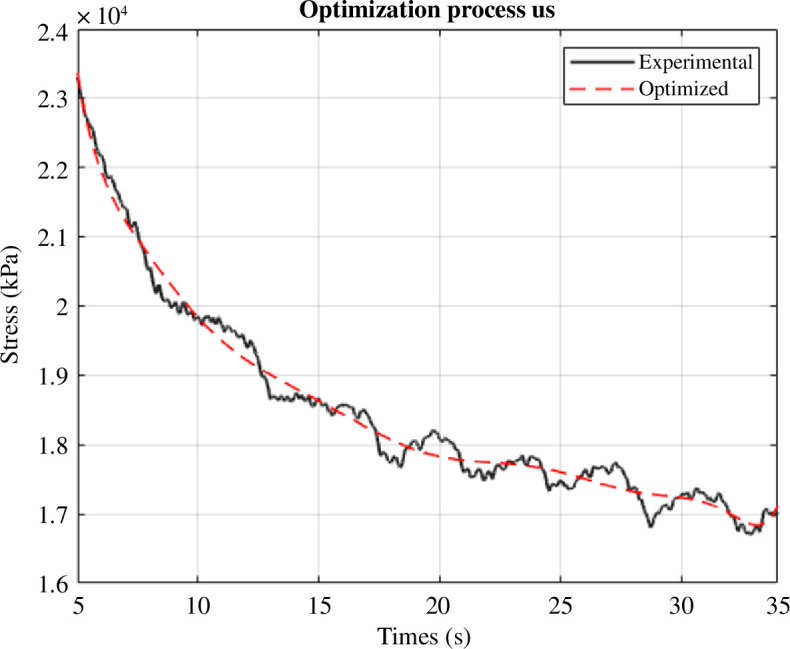
Relaxation experimental curve fitted by the method presented in §2.3.1.

The following equation (2.4) allowed us to obtain the parameters of the skin from the Prony parameters, minimizing the error between the estimated curve and the experimental curve (figure 3), according to Serra-Aguila *et al.* [26]:


(2.4) $$\left\{ \begin{array}{l} p_{i}=\frac{E_{i}}{E_{0}\;+\;E_{1}\;+\;E_{2}}\\ \tau_{i}=\frac{\eta_{i}}{E_{i}} \end{array}\right.$$

where $E_{i}$ is the elastic character of the skin and $\eta_{i}$ is the viscous character of the skin.

#### 2.3.2. Elastic modulus analysis

An original MATLAB® code was developed to calculate the elastic moduli $E_{1}\wedge E_{2}$ of the skin in extension. From the stress–displacement curves obtained by the relaxation tests, the algorithm searches for the two linear parts of the curve. The fitlm function in MATLAB® is used to calculate the slopes corresponding to the $E_{1}\wedge E_{2}$ moduli according to Hooke’s law. This function is based on a linear regression model applied only to the linear parts of the curve.

### 2.4. Image analysis: model for calculating deformations

To calculate the deformation state layer by layer, we used a statistical method developed by Aüllo-Rasser *et al.* [28]. This method determines the deformation rate between an initial image (corresponding to rest) and after extension.

In this model, deformations are quantified at the microstructure level of the tissue image. The methodology uses the quantification of image centroid frequencies. Centroid spatial frequencies are determined in the frequency domain of the Fourier transform of the image. The centroid measurement involves determining the location of the spectral centre of gravity of the image. Its value corresponds to a frequency (in Hz), around which the energy of the spectrum is equally distributed on both sides. If $f\left(x,y\right)$ is the LC-OCT or ultrasound image, before or after extension, and the Fourier transform of the image $TF\left(f\left(x,y\right)\right)$ is described as


(2.5) $$TF(f(x,y))=\sum_{K=1}^{N}\sum_{l=1}^{M}f(x,y)e^{-j2\pi \left(\frac{ux}{M}+\frac{vy}{N}\right)}$$

where $j^{2}=-1$ and *M* and *N* are the pixel number of the image in both directions x and y .

We denote the spatial frequencies in both the *x* and *y* directions as


(2.6) $$u_{i}=\frac{i}{N\Delta x}\;and\;v_{j}=\frac{j}{M\Delta y}$$

where i and j vary from 1 to *N* and from 1 to *M*, respectively. Δx and Δy are the steps between two pixels in the x and y image directions.

The spectrum of spatial frequencies ranges from low frequencies for

i=1andj=1 , where $u_{1}=\frac{1}{N\Delta x}\;and\;v_{1}=\frac{1}{M\Delta y}$

to high frequencies for

$i=\frac{N}{2}\;and\;j=\frac{M}{2}$ , with $u_{N/2}=\frac{1}{2\Delta x}\;and\;v_{M/2}=\frac{1}{2\Delta y}.$

We quantify the effect of stretching at the microstructure level of the image by determining the centroid frequencies between low and high frequencies, such as


(2.7) $$u_{x}^{c}=\frac{\sum_{i\;=\;1}^{N}\sum_{j\;=\;1}^{N}u_{x}TF(f(x,y))}{\sum_{i\;=\;1}^{N}\sum_{j\;=\;1}^{N}u_{x}TF(f(x,y))}$$

(2.8) $$v_{y}^{c}=\frac{\sum_{i\;=\;1}^{N}\sum_{j\;=\;1}^{N}v_{y}TF(f(x,y))}{\sum_{i\;=\;1}^{N}\sum_{j\;=\;1}^{N}TF(f(x,y))}$$

The centroid wavelengths of the image along the x and y axes are given as $\lambda_{x}^{c}=\frac{2\pi }{u_{x}^{c}}\;and\;\lambda_{y}^{c}=\frac{2\pi }{v_{y}^{c}}$

If we define the wavelengths of the image before stretching as $\lambda_{x0}^{c}$ and $\lambda_{y0}^{c}$ , and the wavelengths of the image after stretching as $\lambda_{x1}^{c}$ and $\lambda_{y1}^{c}$ , the deformations in the x and y directions of the images are defined as follows:


(2.9) $$\varepsilon_{x}=\frac{\lambda_{x1}^{c}-\lambda_{x0}^{c}}{\lambda_{x0}^{c}},\varepsilon_{y}=\frac{\lambda_{y1}^{c}-\lambda_{y0}^{c}}{\lambda_{y0}^{c}}$$

In this study, this approach was then used on each layer of the skin, i.e. epidermis, dermis and hypodermis, in order to calculate the deformation of the skin layer by layer during a surface extension test.

### 2.5. Statistical analysis

The statistical analysis of the results was performed using a Kruskal–Wallis test because the distributions were not normal. This test provided us with a *p*‐value, which indicates with 95% confidence whether the differences were statistically significant. Data analyses were conducted using XLSTAT, an Excel plugin.

## 3. Results

### 3.1. Mechanical properties: elastic modulus and rheological parameters

The relaxation curves allowed us to obtain the different mechanical parameters of the skin by fitting the different parts of the curves: the elastic modulus $E_{1}\wedge E_{2}$ and the rheological parameters.

#### 3.1.1. Elastic moduli

**Figure 4.**
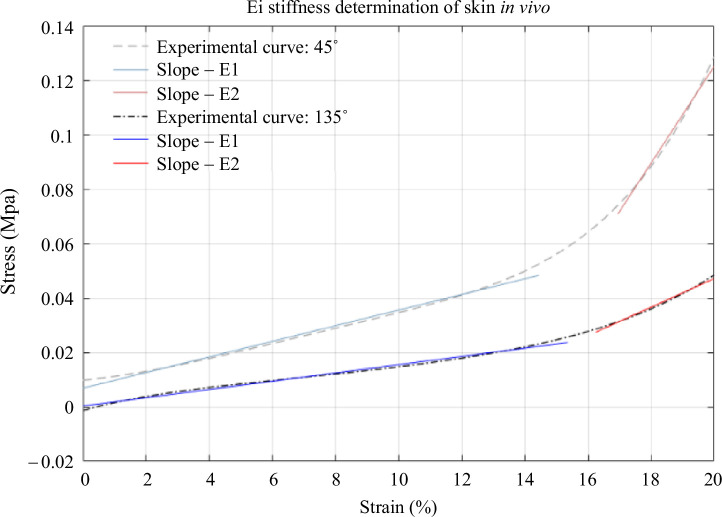
Initial and final slope of a stress–strain curves during loading for two tests: 45° test and 135° test.

The skin is nonlinear under uniaxial extension. Its stress–strain curve is bilinear: phase I and phase III (see figure 4). The slopes of these two phases correspond to the elastic moduli $E_{1}\wedge E_{2}$ quantities that characterize the stiffness of the material as a function of strain and stress. Figure 4 shows the two slopes in the two directions of loading. Figure 4 shows that the 45° curve is higher than the 135° curve. We can see that the stress for 20% deformation is 0.13 MPa at 45° compared with 0.04 MPa at 135°. Similarly, at 10% of strain, the stress at 45° is 0.03 MPa compared with 0.01 MPa at 135°.

Thus, we can see that the slopes of the curve at 45° are higher than at 135°. Table 1 shows the evolution of the elastic moduli $E_{1}\wedge E_{2}$ between the two directions, 45° and 135°, and between the gender of the subjects. The values are the averages of the six male and six female subjects for each direction.

**Table 1.** Elastic moduli (MPa), depending on gender and direction of loading.

	45°		135°	
	$E_{1}$	$E_{2}$	$E_{1}$	$E_{2}$
men (*n* = 6)	3.6 ± 0.8	25.4 ± 8.9	1.8 ± 0.6	5.6 ± 2.1
women (*n* = 6)	2.5 ± 0.6	20.4 ± 8.6	1.4 ± 0.16	7.1 ± 3.2

For men at 45°, we observed that the $E_{1}$ modulus (3.6 MPa) was approximately 10 times weaker than the $E_{2}$ modulus (25.4 MPa), which corresponds to the bilinear character of the curve (see figure 4). However, at 135°, although the behaviour was the same, the difference was not so significant: $E_{1}$ (1.8 MPa) was two times weaker than $E_{2}$ (5.6 MPa).

It was also observed that moduli $E_{1}\wedge E_{2}$ were higher for the 45° loading direction than for 135°. For instance, we found 25.4 MPa for $E_{2}$ at 45° and 5.6 MPa at 135°; likewise for $E_{1}$ .

The same behaviour could be distinguished for women. For both directions, $E_{2}$ was higher than $E_{1}$ . We also observed that the values for both moduli were higher at 45° than at 135°. For example, we found 20.4 MPa for $E_{2}$ at 45° compared with 7.1 MPa at 135°.

Finally, table 1 shows the difference in mechanical behaviour according to gender. The $E_{1}\wedge E_{2}$ moduli are higher for men than for women. For example, for men, $E_{2}$ at 45° is 25.4 MPa, while for women it is 20.4 MPa. Similarly, at 135°, $E_{1}$ is equal to 1.8 MPa for men, but for women, it is 1.4 MPa. These differences were not significant.

Although $E_{2}$ at 135° is higher for women (7.1 MPa) than for men (5.6 MPa). The trend shows these elastic moduli are on average 1.5 times higher for men than for women.

The error calculations for the elastic moduli confirmed the large interindividual variability caused by the *in vivo* measurement. It would be interesting to increase the number of subjects to ‘smooth out’ the possible variations.

The calculation of the elastic moduli shows us the bilinear character of the skin (by $E_{1}$ < $E_{2}$ , with *p* < 0.05) and its anisotropic character (by E45° > E135°, *p* < 0.05).

#### 3.1.2. Rheological parameters

The fit of the skin relaxation curves *in vivo* led us to calculate its rheological parameters: $E_{i}$ will reflect the elastic character of the skin, while $\eta_{i}$ is a parameter that reflects the viscous character of the skin. These parameters, $E_{0},E_{1},E_{2},\eta_{1}\wedge \eta_{2}$ , reflect the evolution of the skin’s viscoelastic properties. The tests were carried out following the protocol given in §2.2.1 and provided us with information on the evolution of the skin according to the direction of solicitation, age and gender. Tables 2–4 show the rheological parameters averaged across the two groups: ages and gender.

**Table 2.** Evolution of the average rheological parameters *E*_*0*_ *,E*_*1*_∧ *η*_*1*_ between the two directions 45° and 135°, independently of gender.

rheological parameters						
	45°			135°		
	$E_{0}\left(kPa\right)$	$E_{1}\left(kPa\right)$	$\eta_{1}\left(Pa\;s\right)$	$E_{0}\left(kPa\right)$	$E_{1}\left(kPa\right)$	$\eta_{1}\left(Pa\;s\right)$
average for men and women combined (*n* = 12)	680.4 ± 222.3	1382.5 ± 730.4	890.3 ± 302.2	209.6 ± 113.0	417.2 ± 568.3	172.1 ± 101.0

**Table 3.** The impact of age on the biomechanical parameters of the skin.

rheological parameters						
	45°			135°		
	$E_{0}\left(kPa\right)$	$E_{1}\left(kPa\right)$	$\eta_{1}\left(Pa\;s\right)$	$E_{0}\left(kPa\right)$	$E_{1}\left(kPa\right)$	$\eta_{1}\left(Pa\;s\right)$
subject <30 years (*n* = 6)	719.4 ± 178.6	1450.7 ± 672.8	1121.0 ± 252.11	276.6 ± 113.4	750.7 ± 640.3	184.8 ± 90.1
subject >30 years (*n* = 6)	588.8 ± 241.7	850.4 ± 472	774.8 ± 243.3	170.0 ± 83.5	328.3 ± 383.0	176.3 ± 110.6

**Table 4.** The impact of gender on the biomechanical parameters of the skin.

rheological parameters						
	45°			135°		
	$E_{0}\left(kPa\right)$	$E_{1}\left(kPa\right)$	$\eta_{1}\left(Pa\;s\right)$	$E_{0}\left(kPa\right)$	$E_{1}\left(kPa\right)$	$\eta_{1}\left(Pa\;s\right)$
men (*n* = 6)	727.4 ± 263.9	1241.3 ± 496.6	988.2 ± 343.4	224.1 ± 119.9	497.7 ± 501.9	137.2 ± 74.1
women (*n* = 6)	580.8 ± 135.85	1059.8 ± 902.8	907.7 ± 248.1	222.4 ± 105.5	581.4 ± 624.9	224.0 ± 105.6

Table 2 shows the evolution of the average rheological parameters $E_{0},E_{1}\wedge \eta_{1}$ between the two directions 45° and 135°, independently of gender. It can be seen in table 2 that for each rheological parameter, the values are higher for 45° compared with 135°. For instance, $E_{0}$ (respectively, $\eta_{1}$ ) at 45° is equal to 680.4 kPa (respectively, 890.3 Pa s), whereas at 135°, it is equal to 209.6 kPa (respectively, 172.1 Pa s). A similar tendency is observed for $E_{1}$ . The differences between the two directions are statistically significant for the three parameters studied (*p* < 0.05).

Table 3 shows the influence of age on the biomechanical properties of the skin. First, for the 45° direction the values are also higher than 135° regardless of age. It can be seen again that the parameters are higher at 45°. At 135°, for subjects <30 years old, $E_{0},E_{1},\wedge \eta_{1}$ are 276.6 kPa, 750.7 kPa and 184.8 Pa s, respectively, whereas at 45°, $E_{0},E_{1},\wedge \eta_{1}$ are 719.4 kPa, 1450.7 kPa and 1121 Pa s, respectively. The same phenomenon is observed for subjects less than 30 years old. For example, $E_{0}$ at 45° for a subject more than 30 years old is 588.8 and 170 kPa at 135°.

Table 3 also informs us that the mechanical parameters are higher for younger subjects. We can see that for the 45° direction the results for subjects less than 30 years are 719.4 kPa for $E_{0}$ , 1450.7 kPa for $E_{1}$ and 1121 Pas for $\eta_{1}$ , while for subjects older than 30 years the values are 588.8 kPa for $E_{0}$ , 850.4 kPa for $E_{1}$ and 778.8 Pas for $\eta_{1}.$ The phenomenon is similar for the 135° direction where for subjects less than 30 years and older than 30 years, the values are, respectively, 276.6 and 170 kPa for $E_{0}$ , 750.7 and 328.3 kPa for $E_{1}$ and 184.8 and 176.3 Pas for $\eta_{1}$ .

The differences between the ages are not significant, even though we observe a clear trend for each parameter. An increase in the number of subjects would help confirm the results of this preliminary study.

Table 4 shows that the rheological parameters are higher at 45° than at 135°, independently of gender. For women, at 45°, $E_{0}$ decreases from 580.8 to 222.4 kPa ; for men, $E_{0}$ decreases from 727.4 kPa to 224.1 kPa . The same applies to the other parameters $E_{1}$ and $\eta_{1}$ .

The results show us that men have higher parameters than women at 45° of loading. Indeed, at 45°, the values of $E_{0},E_{1}\wedge \eta_{1}$ are, respectively, 727.4 kPa , 1241.3 kPa and 988.2 Pa s for men and 580.8 kPa , 1059.8 kPa and 907.7 Pas for women. However, at 135°, the difference in the results is not significant, with overlapping values. For men, 224.1 kPa, 497.7 kPa and 137.2 Pa.s are found for $E_{0},E_{1}\wedge \eta_{1}$ and 222.4 kPa, 581.4 kPa and 224 Pa s for women. Similarly, here, the differences are not significant.

#### 3.1.3. The prospects of a medical study

Various parameters influence the mechanical response of the skin, including its state of health. We therefore studied the behaviour of two pathological skins: one with crest and one with heliodermatitis, following the same protocol as presented in §2.2 of this article.

**Figure 5.**
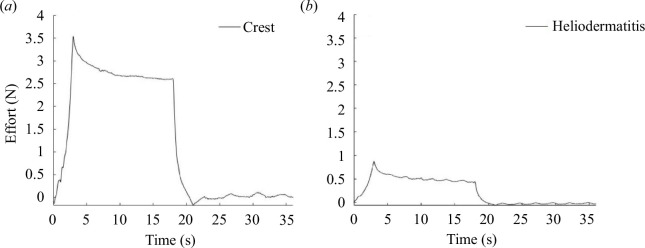
Curves depicting relaxation, as per the procedure described in §2.2, were acquired at a 45° angle, encompassing: (*a*) connective tissue pathology (crest), and (*b*) heliodermatitis (HD), which pertains to skin ageing.

The relaxation curves obtained are shown below in figure 5.

Figure 5 shows that the effort required to apply the 20% extension is not the same on the two curves *a* and *b*. For crest pathology the load $W_{crest}$ is 3.5 N, for heliodermatitis $W_{HD}=1.8\;N$ , whereas for healthy skin, the load is between the two: $W_{sain}=2.7\;N$ .

These differences can also be seen for the elastic moduli in table 5. The hierarchy is indeed the same as for the efforts: $E1_{crest}>E1_{sain}>E1_{HD}$ and $E2_{crest}>E2_{sain}>E2_{HD}$ .

**Table 5.** Elastic moduli of pathological skin and healthy skin at 45°.

	$E_{1}$ (MPa)	$E_{2}$ (MPa)
crest	2.5	10.6
healthy skin	0.8	3.0
heliodermatitis	0.6	2.0

### 3.2. Skin behaviour layer by layer under extension

The coupling between the extension and imaging devices allowed mapping and visualizing the behaviour of the skin from the superficial (epidermis) to the deepest layer (hypodermis/fascia), layer by layer. The aim was to observe if all layers behaved in the same way. The direction of extension was the axis of the arm.

#### 3.2.1. Evolution of the epidermis and superficial dermis

**Figure 6.**
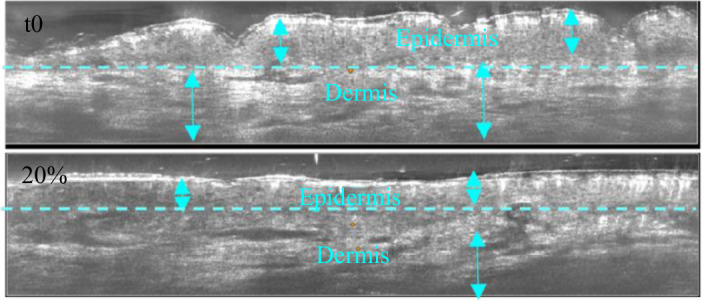
LC-OCT images of the skin from the surface to the superficial dermis (approximately 400 µm) obtained at the University Hospital of Saint-Etienne. At *t*0 and 20% extension. On the forearm (*n* = 1 and man older than 30 years) following the same approach as for the relaxation tests, see protocol given in §2.2.

The use of LC-OCT made it possible to obtain images of the epidermis and superficial dermis. Coupled with the extension device, it was observed that these two layers stretched. It was also observed that the epidermis became thinner (see figure 6).

**Figure 7.**
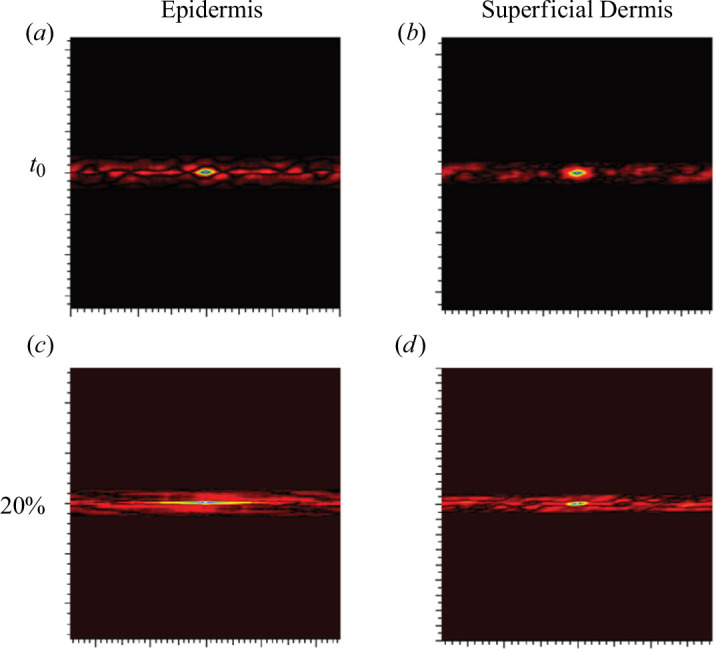
Autocorrelation images of LC-OCT images of the epidermis and superficial dermis. On the forearm (*n* = 1 and man older than 30 years) following the same approach as for the relaxation tests (see protocol given in §2.2.

The autocorrelation of these two images (figure 6) also shows the stretching of these two layers (figure 7). The patterns found in the autocorrelation images (figure 7*a*,*b*) for the epidermis and superficial dermis correspond to the resting state of the skin.

These patterns are found to be highly stretched in the autocorrelation images at 20% extension (figure 7*c*,*d*). This shows the stretching of these skin layers under extension.

#### 3.2.2. Evolution of the dermis, hypodermis and fascia

**Figure 8.**
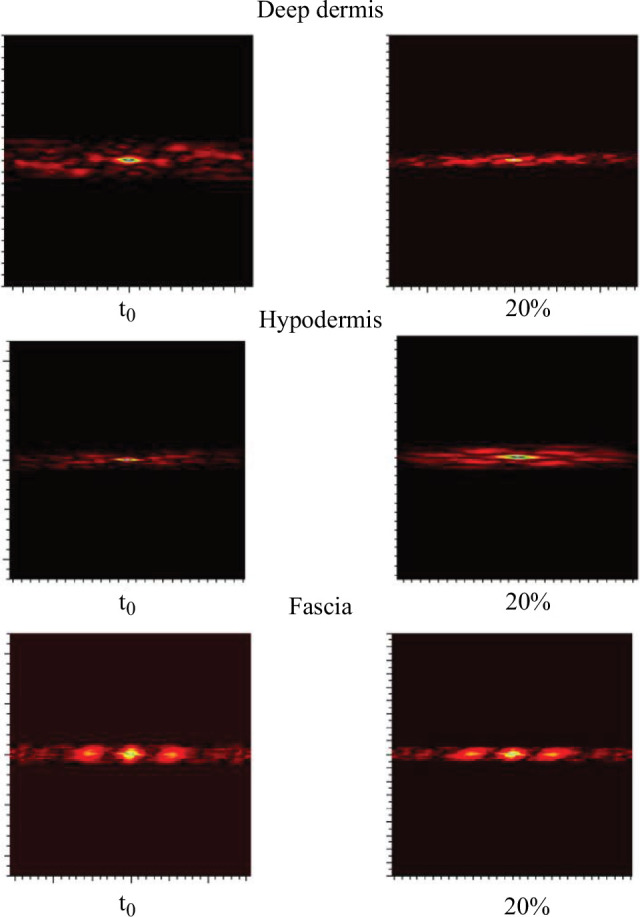
Autocorrelation images of the ultrasound scan images for each layer, deep dermis, hypodermis and fascia. On the forearm (*n* = 1 and man older than 30 years) following the same approach as for the relaxation tests (see protocol given in §2.2).

**Figure 9.**
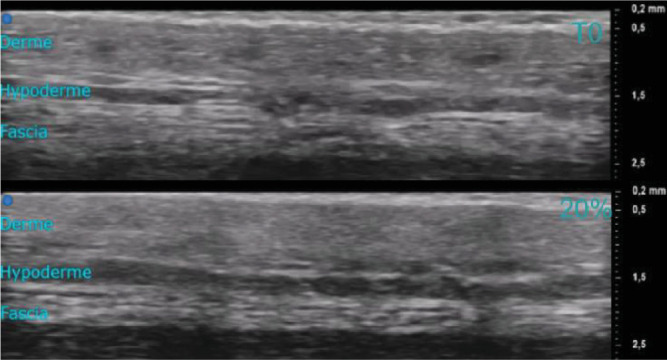
Ultrasound images of the skin during an extension test performed at the University Hospital of Saint-Etienne. The three deep layers of the skin can be seen: dermis, hypodermis and even the fascia at *t*0 and 20% extension. These images correspond to the forearm (*n* = 1 and man older than 30 years) following the same approach as for the relaxation tests (see protocol given in §2.2).

The extension-ultrasound scan coupling (figure 8) allows visualizing the evolution of the deeper layers of the skin (deep dermis, hypodermis or even the fascia). Indeed, these tests show that the skin stretches to the hypodermis. The skin seems to slide over the fascia, which stretches very little. The autocorrelation of the ultrasound (figure 9) scan images of each layer shows the stretching of each layer in the same way as in figure 7.

Figure 9 shows how the deeper layers, such as the deep dermis and hypodermis, deform under the effect of surface extension. The patterns of the deep dermis and hypodermis stretch between *t*0 and 20% extension in the same way as for the more superficial layers. This is clearly seen in figure 9—deep dermis and hypodermis.

In contrast, for the fascia, the patterns deform much less than the skin layers (see figure 9—fascia). The pattern remains almost the same between the two extension states.

#### 3.2.3. Comparison of deformation layer by layer

In summary, all the layers of the skin deformed—stretched—under the effect of surface extension: the epidermis and superficial dermis, the deep dermis and the hypodermis. The fascia, on the other hand, deformed less than the others. But the question that arises is whether all the layers are deformed in the same way, knowing that each layer is different from a structural point of view.

Thus, a statistical method based on the wavelengths of an image (see §2.4) was used to calculate the deformations along $\vec{x}\wedge \vec{y}$ layer by layer using the coupling between the extension and imaging devices.

Table 6 shows the *x*- and *y*-strain ( $\epsilon_{x}\wedge \epsilon_{y}$ ) and Poisson’s ratio ( ν ) calculated from the image correlation algorithm and the LC-OCT and ultrasound scan images for each skin layer, from the most superficial to the deepest. For the measurement of Poisson’s ratio, we rely on the classical relation that determines Poisson’s ratio as a ratio between the deformation along *x* and along *y* such that: $\nu =\frac{-\epsilon_{y}}{\epsilon_{x}}$ . We assume that we remain within the elastic limits.

**Table 6.** Deformation and Poisson’s ratio in extension in the arm axis.

	layers	$\epsilon_{x}$	$\epsilon_{y}$	ν
**extension-LC-OCT**	epidermis	0.22	−0.095	0.43
	superficial dermis	0.37	−0.15	0.41
**extension-ultrasound scan**	deep dermis	0.193	−0.059	0.46
	hypodermis	0.0948	−0.044	0.46
	fascia	0.0618	−0.025	0.4

For the deformation along the *x*-axis, the layer that deforms the most is the superficial dermis: $\epsilon_{x}=$ 0.37. The least deforming part is the fascia: $\epsilon_{x}=$ 0.0618. In the same way as for the deformation along the *y*-axis, the superficial dermis deforms the most: $\epsilon_{y}=-0.15$ and the fascia the least: $\epsilon_{y}=$ −0.025. The hypodermis also deforms very little under extension: $\epsilon_{x}=$ 0.094 and $\epsilon_{y}=$ −0.044. The epidermis and the deep dermis deform more than the hypodermis but less than the superficial dermis. All the layers have the same Poisson’s ratio: about 0.4.

The LC-OCT images allow the calculation of several $\epsilon_{x}$ and $\epsilon_{y}$ deformation values, and the values presented in table 6 are an average.

**Figure 10.**
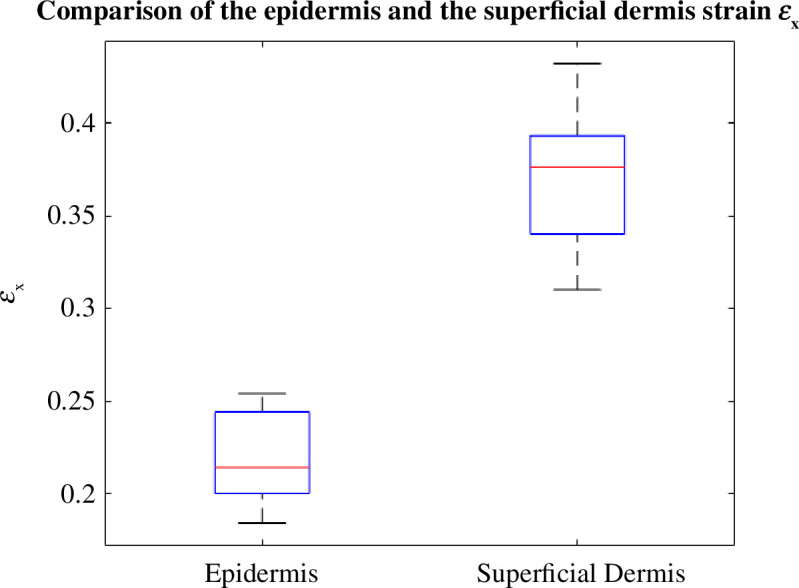
Box plot showing the difference between the $\epsilon_{x}$ deformation of the epidermis and the superficial dermis.

**Figure 11.**
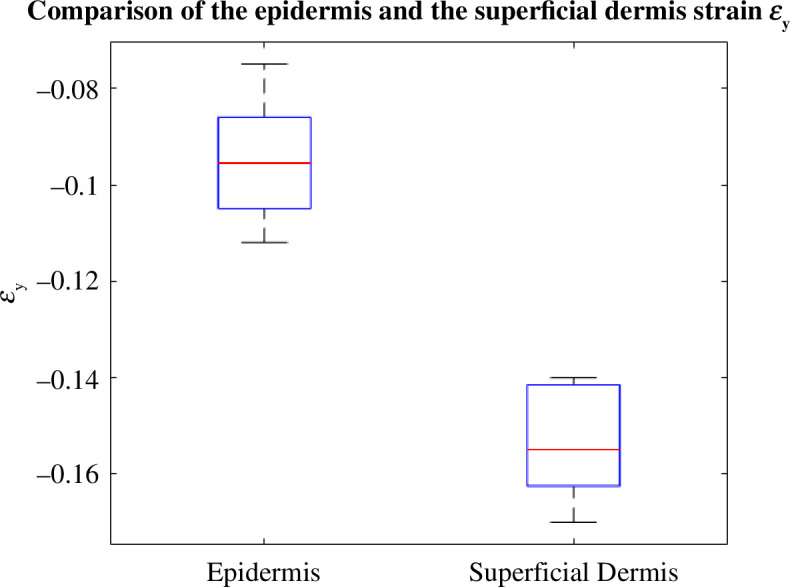
Box plot showing the difference between the $\epsilon_{y}$ deformation of the epidermis and the superficial dermis.

Figure 10 shows the difference in $\epsilon_{x}$ -strain between the epidermis and the superficial dermis. It is clearly seen that all the $\epsilon_{x}$ -strain values of the epidermis are smaller than those of the superficial dermis. For the $\epsilon_{y}$ deformation (figure 11), we can also see that the deformation values of the superficial dermis are higher in the negative than those of the epidermis. To conclude, the superficial dermis deforms more than the epidermis.

**Figure 12.**
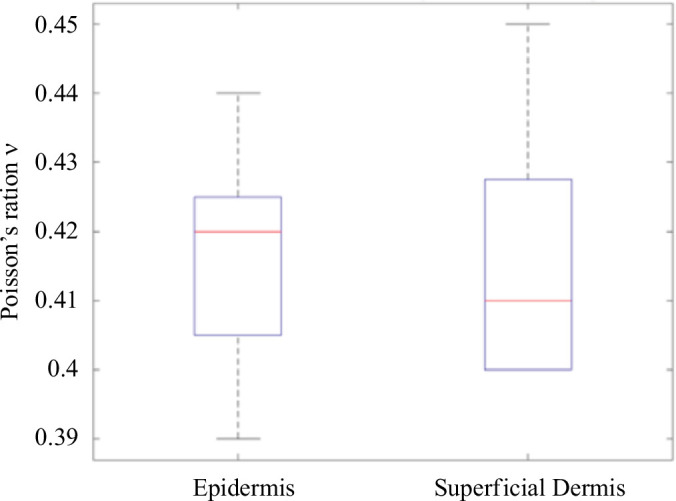
Boxplot of the Poisson coefficient for the epidermis and superficial dermis.

It can be clearly seen in figure 12 that both layers have the same Poisson ratio. The average values for the epidermis and superficial dermis are 0.43 and 0.41, respectively. Deformations were calculated for each layer five times over five different areas of the image. The boxplot makes it possible to observe the dispersion over the five measurements of the same layer while comparing the deformation between the two layers. The results show that the differences in deformation are statistically significant (*p* < 0.05).

### 3.3. Visualization of anisotropy by imaging the skin

**Figure 13.**
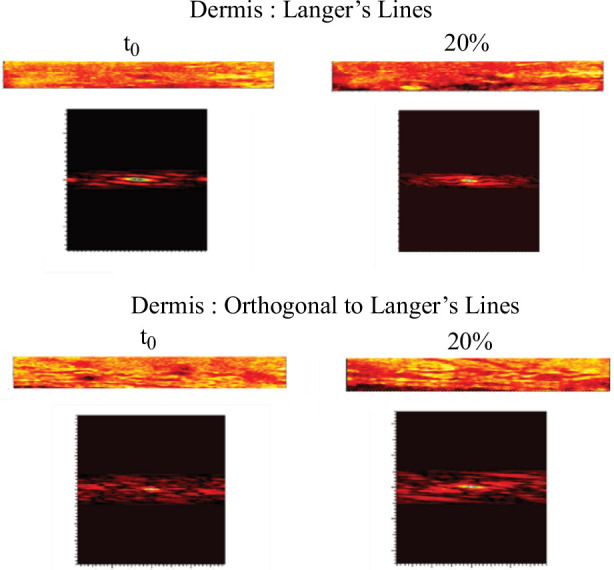
Autocorrelation images of the ultrasound scan images for the dermis. On the forearm in two directions (Langer lines and orthogonal to the Langer lines) (*n* = 1 and man older than 30 years) following the same approach as for the relaxation tests.

The coupling between mechanical testing and imaging in the three directions—Langer line, orthogonal to the Langer lines and arm axis—provides valuable information on the effect of anisotropy on the multi-layer behaviour. We aimed to confirm the results obtained in §3.1, in other words, to observe and confirm the anisotropic nature of the skin through imaging. To do this, we took ultrasound images of the dermis at $t_{0}$ and at 20% extension. As in §3.2.3, from these images, we were able to calculate the deformation of the dermis between the two relevant directions figure 13.

Table 7 shows the difference in deformation between the two directions. It can be seen that in the Langer direction $\epsilon_{x}$ and $\epsilon_{y}$ (respectively, 0.014 and 0.094) are much smaller than in the orthogonal direction (respectively, 0.3 and 0.24). Moreover, $\epsilon_{y}$ is positive in both load directions. Therefore, we observe a difference between these two axes.

**Table 7.** The deformation rate of the dermis in two directions: Langer lines and orthogonal to the Langer line.

	Langer lines		orthogonal to the Langer lines	
**layers**	$\epsilon_{x}$	$\epsilon_{y}$	$\epsilon_{x}$	$\epsilon_{y}$
dermis	0.014	0.094	0.3	0.24

## 4. Discussion

### 4.1. The device developed

One of the main objectives of this study was to characterize the mechanical behaviour of the skin. To study the influence of certain factors on its mechanical parameters, we developed an extension system and calculation tools, and we coupled the system to extension devices. The results we obtained validated this device and the protocols. Indeed, we observed that skin response can be influenced by factors such as age and the direction of solicitation, and we could even observe the evolution of the skin layer by layer during a surface extension. The results were very satisfying and showed the skin’s behaviour when subjected to mechanical stress. Consequently, we can already validate the method developed for this study.

The perspectives are now to study the influence of other factors on skin response, such as pathologies and BMI, and to increase the number of subjects.

### 4.2. Mechanical parameters: elastic moduli and rheological parameters

The values of the elastic moduli $E_{1}\wedge E_{2}$ were between 1.4 MPa and 25.4 MPa. It is interesting to compare these results with the literature. The calculated values were in line with the data found in the literature: from 5 kPa [29] to 6 MPa [30] for $E_{1}$ and from 0.6 MPa [31] to 45 MPa [3] for $E_{2}$ . Our values were within the range of the literature, which reinforced the validation of our device and our protocol. However, our results exhibit relatively high s.d. This is in line with the literature, which provides wide ranges of modulus values because interindividual variability is significant in *in vivo* experiments.

It was noted that the slopes in the 135° direction were lower than those in the 45° direction, which confirmed the anisotropy of the skin. The slopes corresponded to the elastic moduli $E_{1}\wedge E_{2}$ , according to Hooke’s law: σ=Eϵ .

Table 1 shows the calculated elastic modulus values. It was found that the values of the $E_{1}\wedge E_{2}$ moduli at 45° were higher than those calculated at 135°. Tables 2–4 also show that in each case, comparing the ages, gender and average of all the subjects, all the rheological parameters are higher at 45° than at 135°, thereby confirming the strong anisotropy of the skin. Indeed, the fibre network of the dermis is not uniformly distributed in all directions of the skin, which is why the skin is described as anisotropic [32]. The fibre networks are denser in the direction of natural tension. This results in a difference in elongation and tension between the directions of 45° and 135°. It seems that the 45° direction corresponds to the direction where the skin is naturally the most stretched.

Studying the mechanical parameters allowed us to analyse the behaviour of the skin under different conditions: gender, age and direction of different stresses. What we can retain and observe is the influence of these parameters on the biomechanical behaviour of the skin.

Tables 1 and 4 show the effect of gender, and, in general, men do not have the same response as women. At 45°, we observe that the parameters are higher for men. However, this is less clear for a direction of 135°. Table 3 shows that age influences the biomechanical response of the skin. The elastic and viscous parameters are higher for younger subjects. However, these results are only trends, as the differences are not statistically significant. These tests demonstrate that our method is capable of detecting this type of difference. However, it is necessary to increase the number of subjects to validate the proposed hypotheses.

We also observed the influence of pathologies on the mechanical response of the skin. The results obtained are very consistent with the literature and what we know about these pathologies. Indeed, the mechanical results were higher for the patient suffering from the crest, a disease that leads to an excess of fibres and therefore abnormally rigid skin (table 5). In contrast, the patient with heliodermatitis had the lowest mechanical parameters. This is logical since this pathology includes early skin ageing and therefore skin relaxation. Between the two, we find the healthy patient whose skin was neither too rigid nor too relaxed (table 1).

These results are very encouraging for the future. Our method is capable of detecting the influence of pathologies on the biomechanical response of the skin. However, these results need to be confirmed by increasing the number of subjects.

In general, we studied the parameters of gender, age, the direction of solicitation and pathologies, but other cases could be tested such as BMI, lifestyle (sportspersons, smokers, etc.) and phenotype. This first approach using the extension test points to the possibility of starting future tests on a larger panel, on other areas of the body and on pathological skins. The advantages of this type of test are that it is carried out *in vivo*, on the skin in its natural state, and it is non-invasive.

### 4.3. Mechanical behaviour of the skin in depth during extension

Figures 4–8 show us the behaviour of the skin when subjected to extension. We can see that each layer deforms and stretches, from the surface to the hypodermis. On the surface, the microrelief is oriented in the direction of the stress. Deeper down, each layer stretches. The overall behaviour of the skin is therefore to stretch in the direction of stress, from the microrelief to the hypodermis. However, we note that the fascia does not deform very much (figure 9). Therefore, during surface extension, the skin moves by sliding over the fascia, which plays the role of a solid and static structure between the skin and the muscles.

However, because the skin is a non-homogeneous material, its overall behaviour results from the behaviour of all the layers, which are structurally different. That is why this study presented the behaviour of the skin layer by layer, to see if all the layers deform in the same way. Table 6 shows the difference in deformation between the skin layers. Indeed, the epidermis, which is a rigid layer, deforms much less than the dermis, $\epsilon_{x}=0.22\wedge \epsilon_{x}\left(dermis\right)=0.37.$ This is consistent with their composition, as the epidermis is composed of keratinocytes, which are rigid cells, whereas the dermis is composed of elastic fibres. More generally, each layer deforms differently, the reason being their different structures and compositions. The skin exhibits a stiffness gradient, with the epidermis, comprising multiple layers of keratinocytes undergoing desquamation, displaying varying rigidity levels from the outermost layer (stratum corneum, the most rigid) to the dermal–epidermal junction [33]. Our findings reveal that, owing to the presence of the highly rigid stratum corneum, the epidermis experiences less deformation compared with the dermis. This outcome aligns with expectations because the dermis comprises a network of elastic fibres (collagen and elastin) designed to stretch when the skin encounters mechanical stress.

Moreover, we observed that the deformations of the deep layers (hypodermis and deep dermis) were quite small, unlike the more superficial layers. Indeed, as the extension was on the surface, the deformation applied by the extension device should not be the same at depth because of the structures present in the skin and its viscous character, which causes energy dissipation.

Thanks to the coupling of multi-depth imaging (LC-OCT and echography), we were able to determine Poisson’s ratio of the three skin layers and the fascia. The Poisson’s ratio does not change from the epidermis to the fascia. This showed the global character of this parameter. Thus, even if the skin is a non-homogeneous material, its global behaviour corresponds to the coherence between all its layers. Therefore, if all these layers had Poisson’s ratio of 0.4, we can conclude that Poisson’s ratio of the skin is 0.4.

### 4.4. Anisotropy in images

The study of the global behaviour of the skin through the calculation of the viscoelastic parameters showed the strong anisotropy of the skin, between the Langer lines and its perpendicular direction. The question that naturally arose was: what is the influence of the direction of loading from the multi-layer point of view?

Thus, the coupling between the extension test and echography in both directions allowed us to follow the same protocol as in §3.2.3, allowing us to calculate the dermis deformations for each direction of stress. What we observed was that the deformations varied for the three directions. The direction of minimum tension, the Langer lines, deformed less (in depth) than the perpendicular direction. This difference was caused by the different orientations of the fibres.

With these results, we confirm what we found earlier in the study. The skin is strongly anisotropic. Here, we add that this anisotropy also appears deep down, especially in the dermis, where the fibres are located.

**Figure 14.**
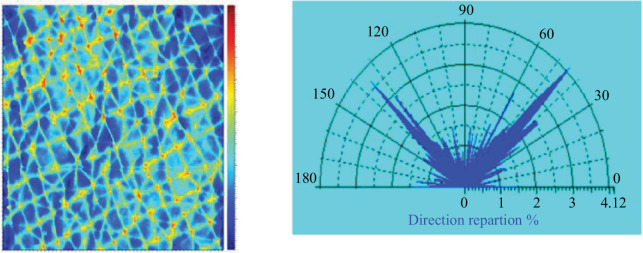
Image (5.5 × 5.5) of a microrelief print at rest, and a study of the orientation of the grooves. *n* = 1, men less than 30 years.

We could see that in depth, the skin can be modelled with the following two families of fibres: those following Langer lines and those perpendicular to these lines. This pattern of two fibre families can also be seen through the analysis of the microrelief. Indeed, the microrelief reflects what is happening deep down in the fibre networks of the dermis [12]. Figure 14 shows the orientation of the skin microrelief, and what we observed are the same directions as visualized in this figure, which shows the two directions of the two families of fibres.

When we stretched the skin in one of these two directions, it behaved like an auxetic material, i.e. when we applied a tensile force the material swelled. However, in the axis of the arm, as we saw above, when the skin was stretched, the layers became thinner ( $\epsilon_{x}>0,\epsilon_{y}<0$ ), because in the other directions, the behaviour was more global and recruited all the fibres during its deformation.

It is generally accepted that collagen lines exhibit a predominant orientation, characterized by Gaussian distributions around this dominant angle, rather than simply being fibres aligned orthogonally to Langer lines [34]. However, in the context of this preliminary study, we have deliberately chosen to focus on two specific orientations. Experimentally, we have identified two major fibre families. Figure 13 shows that by statistically analysing the orientation of the furrows and valleys in the replicated microrelief, we can discern the principal orientations of the dermal fibres. Furthermore, the work of Zahouani *et al*. [12] has demonstrated that the microrelief is a snapshot of the state of the fibre network in depth.

Finally, this pre-study presents a new approach and a new protocol that allows, contrary to the traditional indentation, suction or traction approach, to study the skin’s multi-layer behaviour. This multi-layer approach will allow us to study the behaviour of the skin in depth. Many fields of research are concerned such as cosmetology: to what depth does a moisturizing cream penetrate the skin? Or dermatology: does the presence of certain lesions or pathology in depth such as dermal cysts influence the mechanical behaviour of all the layers in depth? and many others.

This work presents a method that takes into account the real structure of the skin divided into three layers and composed of a network of fibres that is divided into several families.

## 5. Conclusion

To understand and analyse the mechanical behaviour of the skin, we studied the curves by fitting them to a second-order Maxwell model to obtain the rheological parameters and elastic modulus of the skin in all directions of loading, on young and old, female and male subjects. The system was coupled to imaging devices allowing us to study in parallel the behaviour of the microrelief and the skin in depth during extension. Thus, combining the extension system with imaging devices led us to understand both the general behaviour of the skin and its behaviour layer by layer, each layer being different from the others. The results obtained were a map of the mechanical behaviour of the skin that combines parameters and depth information.

In conclusion, this article presented a method for studying the mechanical behaviour of the skin. From a purely mechanical point of view, we worked on the influence of certain parameters on the mechanical response of the skin, leading to the conclusion that the skin can be influenced by the gender and age of the person, as well as by the direction of solicitation and pathologies. This method could be applied to other parameters such as the influence of a cosmetic product and the difference between healthy and pathological skin.

The second part of this work allowed us to understand the behaviour of the skin in depth during an extension. The difference in behaviour layer by layer confirmed the non-homogeneity of the skin structure and the role of the fascia in the skin’s movement. Following image analysis, the extension–imaging coupling allowed calculating the Poisson’s ratio of the skin and the layer-by-layer deformation.

Finally, this article provided the basis for further knowledge of the mechanical behaviour of the skin in extension from the surface to the depth and the influence of certain factors.

## Data Availability

The datasets supporting this article have been uploaded as part of the supplementary material [35].
